# Cystoid Macular Edema Secondary to Acute Retinal Necrosis: The Role of Fundus Fluorescein Angiography in Guiding Treatment

**DOI:** 10.7759/cureus.96108

**Published:** 2025-11-04

**Authors:** Vipin Rana, Ashish Markan, Atul Arora, Manasi Tripathi, Padmamalini Mahendradas, Vikas Sharma, Utkarsh Roodkee, Sourabh Kumar, Ranjit Goenka

**Affiliations:** 1 Ophthalmology, Command Hospital, Kolkata, IND; 2 Ophthalmology, All India Institute of Medical Sciences, New Delhi, IND; 3 Ophthalmology, Postgraduate Institute of Medical Education and Research, Chandigarh, IND; 4 Ophthalmology, IClinix Eye Hospital, New Delhi, IND; 5 Ophthalmology, Narayana Nethralaya, Bengaluru, IND; 6 Ophthalmology, Air Force Central Medical Establishment, New Delhi, IND; 7 Ophthalmology, Base Hospital Delhi Cantt, New Delhi, IND

**Keywords:** acute retinal necrosis, cystoid macular edema, fluorescein angiography, interferon alpha-2b, intravitreal dexamethasone

## Abstract

Cystoid macular edema (CME) is an uncommon but vision-threatening sequelae of acute retinal necrosis (ARN). We report a patient who developed CME following vitreoretinal surgery for ARN-associated retinal detachment. Multimodal imaging, particularly fundus fluorescein angiography (FFA), revealed diffuse capillary leakage suggestive of persistent retinal vascular inflammation. Initial therapy with topical interferon alpha-2b yielded no anatomical or visual benefit. In contrast, intravitreal sustained-release dexamethasone resulted in rapid resolution of CME and significant visual improvement without reactivation of viral retinitis. This case highlights the potential role of FFA in identifying retinal vascular leakage predictive of treatment response, thereby guiding the treatment. Careful monitoring remains essential given the risk of viral reactivation with intravitreal corticosteroids.

## Introduction

Acute retinal necrosis (ARN) is a fulminant herpetic retinitis characterized by peripheral necrotizing retinitis, occlusive vasculitis, and severe intraocular inflammation. Despite advances in antiviral therapy and vitreoretinal surgery, eyes affected by ARN remain vulnerable to delayed complications that may severely compromise vision [1]. Among these, cystoid macular edema (CME) is uncommon but potentially sight-threatening. Its pathophysiology is incompletely understood, likely involving persistent retinal vascular inflammation, immune-mediated injury, and disruption of the blood-retinal barrier, which may be exacerbated by intraocular procedures [2].

Treatment approaches reported in the literature include intravitreal corticosteroids and interferon alpha-2a or 2b, yet no standardized guidelines exist. Interferons are thought to exert antiviral, immunomodulatory, and barrier-stabilizing effects [3-5], whereas corticosteroids suppress cytokine-mediated vascular leakage and inflammation. However, the risk of viral reactivation with corticosteroids complicates therapeutic decisions [6]. Notably, the role of fundus fluorescein angiography (FFA) in post-ARN CME has not been studied. FFA may provide crucial information on vascular leakage, potentially guiding individualized management and optimizing visual outcomes

We present a case of post-ARN CME developing after silicone oil removal and cataract surgery. FFA identified vascular leakage, and the patient responded to treatment with an intravitreal dexamethasone implant.

## Case presentation

A 32-year-old immunocompetent Asian Indian man presented with painless, progressive diminution of vision in his left eye (LE) one month after undergoing silicone oil removal with internal limiting membrane (ILM) peeling. He was a known case of bilateral ARN, diagnosed in March 2024 and confirmed as herpes simplex virus type 2 (HSV-2) positive by aqueous humor polymerase chain reaction (PCR).

The patient had previously undergone pars plana vitrectomy (PPV) with silicone oil injection in April 2025 for ARN-related retinal detachment in the LE, followed by phacoemulsification with posterior chamber intraocular lens (PCIOL) implantation and silicone oil removal in February 2026. The right eye (RE) had inactive peripheral ARN lesions with preserved visual acuity.

**Figure 1:**
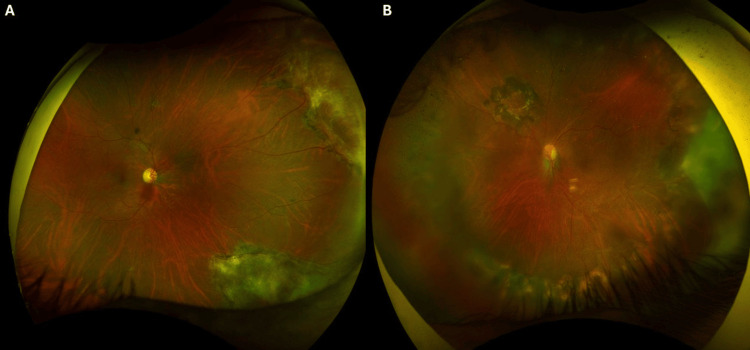
(A) Right eye fundus examination showed circumferential, tongue-shaped, whitish to yellowish pigmented lesions with irregular geographic configuration in the superonasal and inferonasal peripheral retina. The lesions are well demarcated from the surrounding retina. (B) Left eye fundus examination showed multiple confluent, tongue-shaped, whitish to yellowish pigmented lesions with sharply defined margins that were noted circumferentially, predominantly in the temporal quadrant, extending to the inferior and nasal periphery. A retinotomy scar is visible in the superonasal quadrant, approximately two disc diameters from the optic disc, surrounded by barrage laser marks. The foveal reflex appears dull.

At presentation, best-corrected visual acuity (BCVA) was 20/20 in the RE and 20/200 in the LE. Anterior segment examination of the RE was unremarkable, while the LE showed a well-centered PCIOL with a quiet anterior chamber. Fundus examination revealed peripheral healed lesions in the RE (Figure 1A). In the LE, peripheral healed necrotic lesions, a superonasal retinotomy scar, and a dull foveal reflex were noted, with no signs of active retinitis (Figure 1B).

**Figure 2:**
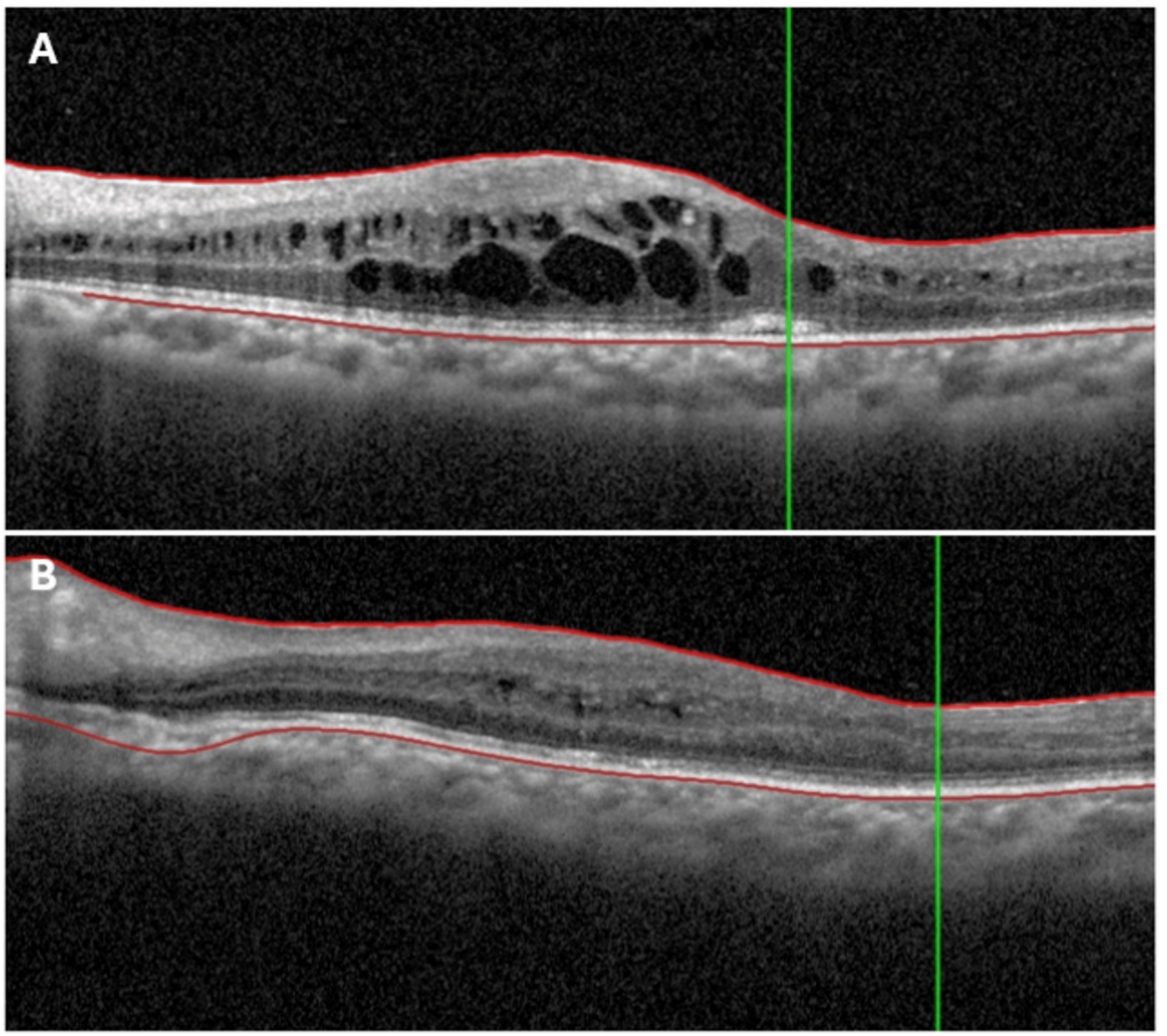
OCT of the left eye showed cystoid macular edema at presentation (A). Ten days later, the OCT demonstrated complete resolution of CME (B). OCT: optical coherence tomography; CME: cystoid macular edema

**Figure 3:**
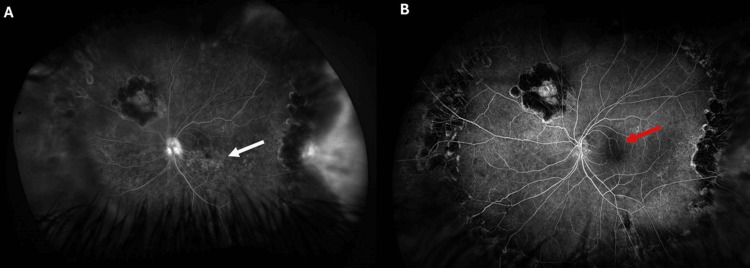
(A) Fundus fluorescein angiography (FFA) of the left eye demonstrating petaloid hyperfluorescence at the fovea (white arrow) with diffuse capillary leakage involving the posterior pole and extending into the periphery, consistent with cystoid macular edema secondary to retinal vascular inflammation. (B) Follow-up FFA two months after intravitreal dexamethasone implant showing a marked reduction in foveal leakage (red arrow) and significant decrease in diffuse capillary leakage across the posterior pole and periphery, with no evidence of retinitis reactivation.

Optical coherence tomography (OCT) (Spectralis, Heidelberg, Germany) of the LE demonstrated CME (Figure 2A). FFA (California, Optos, UK) showed no activity in the RE, but in the LE, it revealed a petaloid leakage pattern at the fovea with diffuse capillary leakage involving the posterior pole and periphery (Figure 3A).

The differential diagnoses considered were pseudophakic CME (Irvine-Gass), post-surgical inflammatory CME following silicone oil removal and ILM peeling, reactivation of viral retinitis, and immune-mediated CME secondary to ARN. Pseudophakic and post-surgical CME usually demonstrate leakage limited to the macula, unlike the widespread diffuse capillary leakage seen here. Viral reactivation was excluded due to the absence of fresh necrotizing lesions or vitritis. Given the angiographic findings and clinical context, the final diagnosis was immune-mediated CME secondary to ARN.

The patient was started on topical interferon alpha-2b drops (1 million IU/mL, six times daily) for two weeks. However, there was no improvement in BCVA or OCT parameters.

Thus, an intravitreal sustained-release dexamethasone implant 0.7 mg (Ozurdex®, Allergan, USA) was administered in the LE. The response was rapid-BCVA improved to 20/60 within five days and further to 20/40 at 10 days, and metamorphopsia resolved. Repeat OCT at day 10 confirmed complete resolution of CME (Figure 2B).

FFA at two months demonstrated a significant reduction in capillary leakage as well as in petaloid leakage in the foveal region (Figure 3B). No reactivation of ARN or development of new infectious retinitis was noted during the eight-month follow-up.

## Discussion

CME is a recognized late complication of ARN, particularly in eyes that have undergone complex vitreoretinal procedures. The pathogenesis is multifactorial, involving persistent low-grade intraocular inflammation, immune-mediated vascular injury, and disruption of the blood-retinal barrier. Surgical interventions such as silicone oil removal and cataract extraction can further exacerbate inflammatory responses, making these eyes vulnerable to delayed macular complications [1,2].

In our patient, CME developed approximately two months after silicone oil removal and cataract surgery. FFA revealed a petaloid leakage pattern at the fovea, characteristic of CME, along with widespread diffuse capillary leakage across the posterior pole. Indocyanine green angiography (ICG) was unremarkable, indicating that the pathology was confined to the retinal vasculature without choroidal involvement. The diffuse leakage suggested active retinal vascular inflammation rather than an isolated Irvine-Gass-type pseudophakic CME.

Topical interferon alpha-2b has been reported as a promising therapy for pseudophakic CME and, in limited reports, post-ARN CME. Its proposed benefits stem from antiviral activity, immunomodulation, and stabilization of the blood-retinal barrier through cytokine downregulation and leukocyte migration inhibition [3-5,7]. Given these reports and the patient’s pseudophakic status, interferon alpha-2b was initiated at six times daily for two weeks. Despite good compliance, there was no anatomical or functional improvement, prompting reassessment of the therapeutic approach.

We postulate that the lack of response to interferon was related to the angiographic profile in our case. The presence of diffuse capillary leakage likely reflected a higher inflammatory burden requiring a more potent and sustained anti-inflammatory intervention. Previous reports of interferon efficacy in post-ARN CME or pseudophakic CME generally did not document diffuse vascular leakage on FFA. This difference in leakage pattern may explain the discrepancy in treatment outcomes.

An intravitreal dexamethasone implant (Ozurdex®) was selected as second-line therapy. Corticosteroids exert broad anti-inflammatory effects, including suppression of pro-inflammatory cytokines, inhibition of leukocyte infiltration, and restoration of vascular endothelial integrity [8,9]. In our patient, the response was rapid and profound-visual acuity improved within five days, metamorphopsia resolved, and repeat FFA at two months showed a marked reduction in leakage. This reinforces the value of intravitreal corticosteroids in cases with extensive retinal vascular inflammation.

However, the use of intravitreal corticosteroids in eyes with a history of herpetic retinitis carries potential risks, including reactivation of ARN and opportunistic infections such as cytomegalovirus (CMV) retinitis [6,10]. Although antiviral therapy was appropriately administered during the acute phase, long-term prophylaxis was not continued thereafter. While prolonged oral valaciclovir (500 mg-1 g twice daily) is often recommended for several months to a year to reduce recurrence risk, especially in bilateral ARN, evidence on its optimal duration remains limited, and practice varies [11]. In our case, despite discontinuation of prophylaxis, no viral reactivation occurred during eight months of follow-up. Although no reactivation occurred in this patient during follow-up, careful monitoring and patient counseling are essential when considering corticosteroid therapy in this setting.

## Conclusions

This case underscores the importance of multimodal imaging in guiding therapeutic decisions for post-ARN CME. FFA not only confirmed the diagnosis but also provided prognostic information that helped predict response to therapy. We suggest that diffuse vascular leakage on FFA may serve as a biomarker for poor response to topical interferon alpha-2b and may favor early initiation of intravitreal corticosteroid therapy. Although systemic corticosteroids are conventionally considered before local steroid delivery, in this case, they were deliberately avoided to minimize systemic immunosuppression and potential reactivation risk. Instead, a localized intravitreal sustained-release dexamethasone implant was preferred to achieve the targeted anti-inflammatory effect with minimal systemic exposure. Prospective studies are needed to validate this approach and refine treatment algorithms for post-ARN CME.
